# The Effect of Fixture Congestion on Performance During Professional Male Soccer Match-Play: A Systematic Critical Review with Meta-Analysis

**DOI:** 10.1007/s40279-020-01359-9

**Published:** 2020-10-17

**Authors:** Ross Julian, Richard Michael Page, Liam David Harper

**Affiliations:** 1 Department of Neuromotor Behavior and Exercise, Institute of Sport and Exercise Sciences, University of Muenster, 48149 Muenster, Germany; 2 School of Sport and Exercise, University of Gloucestershire, Gloucestershire, GL50 2RH UK; 3 Department of Sport and Physical Activity, Edge Hill University, St. Helens Road, Ormskirk, Lancashire L39 4QP UK; 4 School of Human and Health Sciences, University of Huddersfield, Huddersfield, HD1 3DH UK

## Abstract

**Background:**

Fixture congestion (defined as a minimum of two successive bouts of match-play, with an inter-match recovery period of < 96 h) is a frequent and contemporary issue in professional soccer due to increased commercialisation of the sport and a rise in the number of domestic and international cup competitions. To date, there is no published systematic review or meta-analysis on the impact of fixture congestion on performance during soccer match play.

**Objective:**

We sought to conduct a systematic review and meta-analysis of the literature related to the effects of fixture congestion on physical, technical, and tactical performance in professional soccer match-play.

**Methods:**

Adhering to PRISMA guidelines and following pre-registration with the Open Science Framework (https://osf.io/fqbuj), a comprehensive and systematic search of three research databases was conducted to identify articles related to soccer fixture congestion. For inclusion in the systematic review and meta-analysis, studies had to include male professional soccer players, a congestion period that contained two matches ≤ 96 h, and have outcome measures related to physical, technical or tactical performance. Exclusion criteria comprised non-male and/or youth players, data that only assessed impact of congestion on injury, used simulated protocols, or were grey literature, such as theses or dissertations.

**Results:**

Out of sixteen articles included in the systematic review, only five were eligible for the meta-analysis, and the only variable that was measured consistently across studies was total distance covered. Fixture congestion had no impact on total distance covered [*p* = 0.134; pooled standardized mean difference; Hedge’s *G* = 0.12 (− 0.04, 0.28)]. Between-study variance, heterogeneity, and inconsistency across studies were moderate [Cochrane’s *Q* = 6.7, *p* = 0.150, *I*^2^ = 40.7% (CI 0.00, 93.34)]. Data from articles included in the systematic review suggest fixture congestion has equivocal effects on physical performance, with variation between studies and low quality of research design in some instances. Tactical performance may be negatively impacted by fixture congestion; however, only one article was identified that measured this element. Technical performance is unchanged during fixture congestion; however, again, research design and the sensitivity and relevance of methods and variables require improvement.

**Conclusion:**

Total distance covered is not impacted by fixture congestion. However, some studies observed a negative effect of fixture congestion on variables such as low- and moderate-intensity distance covered, perhaps suggesting that players employ pacing strategies to maintain high-intensity actions. There is a lack of data on changes in tactical performance during fixture congestion. With ever increasing numbers of competitive matches scheduled, more research needs to be conducted using consistent measures of performance (e.g., movement thresholds) with an integration of physical, technical and tactical aspects.

## Key Points

**Table:** 

Results of the meta-analysis indicate that fixture congestion has no impact on total distance covered. However, other physical performance variables, such as low- and moderate-intensity distance covered, may be negatively impacted during congested periods.
Tactical performance may be negatively impacted by fixture congestion, with decreased synchronisation between players. However, these findings are from only one article; as such, more research is required on this area. Integration of team behaviour (e.g., team synchrony) with contemporary measures of technical and physical performance is warranted.
There is a lack of consistency between studies measuring the impact of fixture congestion on performance. Fixture congestion is a contemporary and concerning issue (including to the players themselves) and more research is required to elucidate changes in performance.

## Introduction

It is possible for soccer teams to compete in 50–80 matches during a ~ 40-week competitive season, thus regularly playing two matches per week, with some teams completing as many as three matches in a weekly microcycle [1–3]. Contemporary congested match scheduling can be attributed to a number of factors, such as, but not limited to, the increased commercialisation of the sport and the subsequent manipulation of match scheduling in favour of TV revenue, inclement weather conditions and, thus, the postponement of matches, and increased numbers of domestic and international cup competitions.

In a recent survey of 543 elite professional players by the World Players’ Union (FIFPro), 35–40% of players believe that they are currently competing in too many competitive matches, and thus are receiving an inadequate number of days for recovery [4]. In concordance with this perception, previous research has observed that some players, although potentially dependent on playing standard, may still not be 100% recovered in the 72 h following a competitive match [5]. For example, measures of sprint and countermovement jump performance [6–8], thigh muscular isokinetic torque [6, 8], and biochemical markers, such as creatine kinase and uric acid [6, 8], remain significantly impaired when compared to baseline levels at ≥ 72 h post match. In addition, Brownstein et al. [9] identified that players’ perceptions of fatigue persisted 72 h post match play. It should also be acknowledged that as is the case with applied sport and the completion of congested schedules (a minimum of two successive bouts of match-play, with an inter-match recovery period of < 96 h), players who are often not fully recovered are required to compete in a subsequent match. The physical and mental demands of these matches can also be further exacerbated by additional confounding factors, such as travelling to and from away matches [10, 11], with two-thirds of the players surveyed suggesting that travel is a potential factor that limits their recovery [4]. Furthermore, during these congested periods, it is common for matches to be played during the evening, as such, the timing of matches may affect indices of sleep which may then further exacerbate the recovery time course of a player [12].

Although the rotation of squads may prevent some players from competing in congested schedules, a study conducted with a French Ligue 1 (highest professional league in France) club identified that ~ 25–40% of players are required to complete all matches during a two- or three-match microcycle [13]. However, this may be higher in certain clubs, particularly in the lower tiers of domestic leagues where fixture congestion is regularly observed. It is for this reason that insufficient recovery between successive matches and the occurrence of congested fixture periods has been previously suggested as a factor that affects performance. As such, it is of importance to fully understand the magnitude of the effect a congested schedule has on match performance.

Although prolonged physical recovery can in turn lead to residual fatigue and consequently impair physical performance, there has been suggestion that other elements of performance may be affected. One of the main determinants of successful soccer performance is technical ability, which encapsulates, inter alia, passing, shooting and dribbling. Although it has been suggested that physical fatigue which occurs throughout a match can lead to a reduction in successful technical performance, there has been few studies to observe the effect of a congested schedule (which may include residual fatigue) on technical performance [3, 14, 15]. Although the limited literature suggests that a congested schedule does not affect technical performance, it is important to systematically assess whether the literature confirms this proposal and to what magnitude. Therefore, a comprehensive overview of the literature is necessary, to identify what technical performance parameters might be affected. Furthermore, previous research has suggested that congested schedules may affect tactical performance [16]. This may be due to factors such as mental fatigue, with players attempting to cognitively process multiple different instructions and events over a relatively short period of time [17]. Moreover, as mentioned previously, during periods of congestion, teams are regularly rotated and, therefore, the tactical cohesion of the team might be disrupted. As such, further information is required to understand the effect of congested schedules on tactical performance.

Accordingly, there is a need for research to robustly assess the current literature and quantify the effect of a congested schedule on physical, technical and tactical performance. Although elements of previous literature have been reviewed in an opinion piece by Carling et al. [18], a systematic review has not been conducted in this area. Moreover, since the publication of Carling et al. [18], there has been a considerable number of articles published which are specific to this area. Therefore, the purpose of this systematic review is to identify whether a congested schedule affects physical, technical or tactical performance. Moreover, a meta-analysis will be conducted to identify what physical performance parameters are affected by congested schedules. Additionally, this review aims to identify areas for future research and directions in the topic of fixture congestion and its effects on performance.

## Methods

A systematic review and meta-analysis were conducted to evaluate the impact of fixture congestion on in-match physical, technical and tactical performance. The review and meta-analysis were conducted and reported in accordance to the PRISMA (Preferred Reporting for Systematic Reviews and Meta-Analyses) statement (https://www.prisma-statement.org/). The protocol was pre-registered on the Open Science Framework prior to full searches and analysis was undertaken (https://osf.io/fqbuj).

### Search Strategy

Literature searches of PubMed, MEDLINE, and Scopus were undertaken to identify suitable journal articles. All searches were conducted in September 2019 by two of the authors (LDH and RJ). Searches included the following keywords as search terms: “soccer”, or “football” in combination with “fixture congestion”, “congestion”, “congested”, and “match congestion”. Furthermore, reference lists of acquired articles were checked for relevant studies and any articles that were known to the authors were also included. All articles were saved in a reference manager software (EndNote X9, Thomson Reuters©, New York, NY, USA). Following the removal of duplicates, the titles and abstracts of the remaining articles were independently screened for relevance. Finally, the remaining full texts were examined by the two aforementioned authors based upon the inclusion and exclusion criteria, outlined in Sect. 2.2. If there were any discrepancies between authors, then a third author (RMP) checked the relevant article and a consensus decision was reached.

### Selection Criteria

#### Inclusion

To be considered for the present systematic review article, papers needed to fulfil the following criteria: (1) original article written in English; (2) abstracts available for screening; (3) relevant data concerning the effect of fixture congestion on physical and/or technical and tactical performance during soccer match-play; (4) minimum of two matches ≤ 96 h; (5) included male soccer players. There were no restrictions in terms of publication date.

#### Exclusion Criteria

Manuscripts were omitted from the review if they violated any of the following criteria: (1) inclusion of female soccer players; (2) assessed the effects of congestion on youth soccer players; (3) data that only assessed the impact of congestion on injury; (4) used protocols which simulate the demands of soccer match play; (5) published in the following formats: grey literature, such as theses and dissertations (conference proceedings were included if sufficient detail was reported to enable a full quality assessment), as well as reviews, systematic reviews and meta-analyses.

### Assessment of Quality of Methodologies of Studies

The methodological quality of the studies included in this systematic review was evaluated in accordance with previously published work [19], based on the original version developed by Law et al. [20]. The quality of the included methodologies was assessed using a 16-item assessment tool created for quantitative studies; the specific items can be found in Table 1.

**Table 1:** Quality Criteria from Sarmento et al. [15], adapted from Law et al. [16]

Q1	Was the study purpose stated clearly?
Q2	Was relevant background literature reviewed?
Q3	Was the design appropriate for the research question?
Q4	Was the sample described in detail?
Q5	Was sample size justified?
Q6	Was informed consent obtained? (if not described, assume No)
Q7	Were the outcome measures reliable? (if not described, assume No)
Q8	Were the outcome measures valid? (if not described, assume No)
Q9	Was the method described in detail?
Q10	Were results reported in terms of statistical significance?
Q11	Were the analysis methods appropriate?
Q12	Was the importance for practice reported?
Q13	Were any drop-outs reported?
Q14	Were conclusions appropriate given the study methods?
Q15	Are there any implications for practice given the results of the study?
Q16	Were limitations of the study acknowledged and described by the authors?

For each item, quality was rated as 1 (meets criteria), 0 (does not meet criteria) or N/A (not applicable). The final score of each research paper corresponded to the sum of every score in a given article divided by the total number of scored items for that specific research design and expressed as a percentage. Furthermore, methodology quality thresholds were implemented and classified as follows: (1) low (≤ 50%); (2) good (51–75%); and (3) excellent (> 75%) as per [19]. The quality of each methodology was assessed by two authors (LDH and RJ). To ensure there was an acceptable level of inter-rater agreement, Cohen’s kappa coefficient (*ĸ*) was calculated.

## Meta-analysis

A meta-analysis was undertaken to assess the effect of fixture congestion on total distance covered during match-play. Total distance covered was the only variable included in the meta-analysis due to it being the only variable that was measured and reported with enough similarity between studies (*n* = 5). All other variables were not measured in a homogenous way between studies, precluding a meta-analysis to be undertaken. A meta-analysis using random effects was conducted using the “metafor” package in *R* (R Foundation for Statistical Computing, Vienna, Austria. URL: https://www.r-project.org/ [21]). Standardized mean differences (SMD; Hedges’ *G*) for the five studies included in the meta-analysis were calculated using the inverse variance method, with statistical heterogeneity calculated using the *I*^2^ statistic. Low, moderate and high risk of heterogeneity thresholds were set at < 25%, 25–75%, and > 75%. To visualise potential funnel plot asymmetry, standard errors were plotted against Hedges’ *G* values. Furthermore, Duval and Tweedie’s Trim and Fill method was used to assess funnel plot asymmetry. Egger’s regression test was not used due to the number of studies being below 10 [22]. Data used in the meta-analysis are publicly available at https://osf.io/2q6aj/.

## Results

**Fig. 1:**
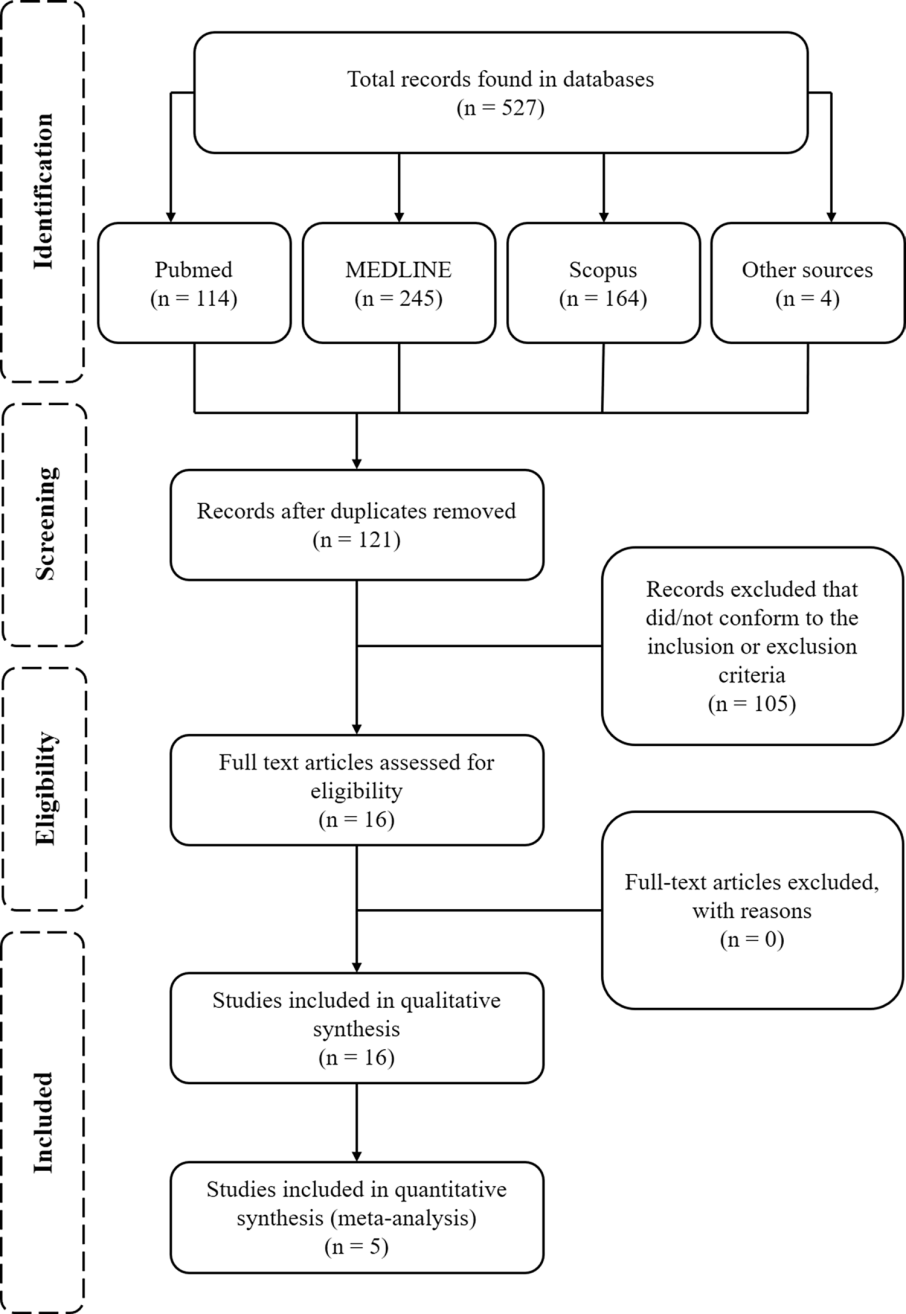
PRISMA flow diagram of the process used in selection of the journal articles included in the systematic review and meta-analysis

An initial search yielded 527 records, with 406 duplicates and, thus, 121 individual records. Following title and abstract inspection, 105 articles were deemed irrelevant, leaving 16 articles eligible for full-text screening. Following full-text screening, all 16 articles were included in the systematic review, five of those included in the meta-analysis. See Fig. 1 for the PRISMA flow diagram. Studies that met the inclusion criteria for the review are presented in Table 2, alongside their quality assessment ratings. The list of studies that were excluded is publicly available at https://osf.io/pcqu3/.

**Table 2:** Summary of studies accompanied by the quality criteria score, investigating the in-match physical performance response during periods of fixture congestion. ↓ and ↑, denotes significant reductions or significant increases in outcome measures, respectively

References	Participants	Match data collection methods	Fixture congestion scenario	In-match outcome measures	Main findings	Quality score (%)
Odetoyinbo et al. [32]	16 elite outfield players from 4 teams in England (FB *n* = 3, CB *n* = 7, CM *n* = 3, WM *n* = 1, FWD *n* = 2)	Semi-automated video system (ProZone)	3 successive matches in 5 days (2 days between matches 1 and 2, and 3 days between matches 2 and 3)	TD and distance covered, frequency and time spent in each locomotive activity. HI distance when the player’s own team is in possession, HI distance when player’s own team is without possession, HI distance when the ball is out of play, recovery time (average time in between HI activity), distance covered per minute of match, average speed, top speed, relative intensity (number of high intensity activities/time) Sprint (≥ 7.0 m^.^s^−1^) HI (> 5.5 m^.^s^−1^) HIR (5.5–6.9 m^.^s^−1^) Run (4.0–5.4 m^.^s^−1^) Jog (2.0–3.9 m^.^s^−1^) Walk (0.2–1.9 m^.^s^−1^) Stand (0–0.1 m^.^s^−1^)	↓ HI distance when team is in possession and when ball was out of play during match 3 vs. match 1 ↓ walking distance match 3 vs. match 1	78.6
Dupont et al. [36]	32 elite outfield players playing for the same Scottish club	Semi-automated video system (Amisco)	1 match microcycles vs 2 match microcycles with ≤ 4 days between match 1 and match 2	TD, HI distance, sprinting distance, frequency of sprints Sprint (> 24 km·h^−1^) HI (19–24 km·h^−1^)	No effect	66.7
Rey et al. [35]	42 elite outfield players from the same Spanish club (FB *n* = 9, CD *n* = 17, CM *n* = 9, WM *n* = 2, FWD *n* = 5)	Semi-automated video system (Amisco)	2 successive matches with 3 days between matches	TD and distance covered in each locomotive activity. Frequency of HIR and sprints, recovery times, top and average speed Sprint (> 23 km·h^−1^) HIR (19.1–23.0 km·h^−1^) MIR (14.1–19.0 km·h^−1^) LIR (11.1–14.0 km·h^−1^) Stand, walk, jog (0–11 km·h^−1^)	No effect	40.0
Carling and Dupont [1]	7 professional midfield (central and wide) players from the same French club	Semi-automated video system (Amisco)	3 successive matches in ≤ 7 days	TD, HIR, TD when individual in possession of the ball, peak period HIR HIR (≥ 14.4 km·h^−1^)	No effect	71.4
Lago-Penas et al. [23]	23 elite outfield players from the same Spanish club (FB *n* = 5, CD *n* = 5, CM *n* = 5, WM *n* = 4, FWD *n* = 4)	Semi-automated video system (Amisco)	1 match vs. 2 match weekly microcyles	TD, distance covered frequency and time spent in each locomotive activity Sprint (> 23 km·h^−1^) HIR (19.1–23.0 km·h^−1^) MIR (14.1–19.0 km·h^−1^) LIR (11.1–14.0 km·h^−1^) Stand, walk, jog (0–11 km·h^−1^)	No effect	80.0
Carling et al. [2]	19 elite outfield players from the same French club	Semi-automated video system (Amisco)	8 successive matches in a 26-day period	Relative TD, light-intensity, LIR, MIR, HIR, and TD in individual ball possession HIR (> 19.1 km·h^−1^) MIR (14.1–19.0 km·h^−1^) LIR (11.1–14.0 km·h^−1^) Light-intensity (0–11 km·h^−1^)	Main effect for differences in TD and light-intensity ↑ TD in matches 4 and 7 compared to 2 and 3 ↑ light-intensity in matches 4 and 8 compared to matches 1, 2, 3, 5 and 6 and 3, respectively	93.3
Dellal et al. [3]	16 elite outfield players from the same French club	Semi-automated video system (Amisco)	3 instances of 6 consecutive matches separated by 3 days (instance 1, 5 players; instance 2, 6 players; instance 3, 5 players)	TD and distance covered in each locomotive activity HIR (> 21.0 km·h^−1^) MIR (18.1–21.0 km·h^−1^) LIR (12.1–18.0 km·h^−1^) Walking and light intensity (0–12.0 km·h^−1^)	No effect	93.3
Andrzejewski et al. [14]	11 professional players from the same Polish club (FB *n* = 2, CD *n* = 3, CM *n* = 2, WM *n* = 2, FWD *n* = 2)	Semi-automated video system (Amisco)	1 vs 2 match weekly microcycles	TD, distance covered in each locomotive activity, frequency of HI and sprinting, recovery time, average and top speed Sprint (≥ 24 km·h^−1^) HIR (21.0–24.0 km·h^−1^) Fast running (17.0–21.0 km·h^−1^) Running (14.0–17.0 km·h^−1^) Slow running (11.0–14.0) Stand, walk, jog (0–11 km·h^−1^)	↑ TD, slow running, running, fast running in match 3 vs. match 1 ↓ standing, walking, jogging in matches 2 and 3 vs. match 1	60.0
Djaoui et al. [24]	16 international players from the same French club (FB *n* = 2, CD *n* = 3, CDM *n* = 3, WM *n* = 3, CAM, *n* = 2, FWD *n* = 3)	Semi-automated video system (Amisco)	4 periods of 1 vs. 2 match weekly microcylces (period 1, 6 matches in 21 days; period 2, 7 matches in 21 days; period 3, 7 matches in 22 days; period 4, 6 matches in 24 days)	TD and distance covered in each locomotive activity Maximal (> 27.0 km·h^−1^) Sub-maximal (> 25.0–27.0 km·h^−1^) VHIR (> 23.0–25.0 km·h^−1^) HIR (> 21.0–23.0 km·h^−1^) Sustained cruising (> 18.0–21.0 km·h^−1^) Light (< 12 km·h^−1^)	No global effect ↓ light intensity for CB and CDM during 1 match microcycles	60.0
Folgado et al. [16]	23 professional players from the same English club	Semi-automated video system (ProZone)	3 successive matches with 3 days between matches	TD and distance covered in each locomotive activity VHIR (> 19.8 km·h^−1^) HIR (14.4–19.7 km·h^−1^) MIR (3.6–14.3 km·h^−1^) LIR (0.0–3.5 km·h^−1^)	No effect	60.0
Mohr et al. [38]	20 players playing in the top three tiers of soccer (country and league not specified)	GPS devices (GPSport 15 Hz)	3 successive matches (3 days between matches 1 and 2; 4 days between matches 2 and 3)	TD and distance covered in HI and sprinting, peak 5-min distance, peak speed, frequency of ACC, DEC and impacts Sprint (> 22 km·h^−1^) HI (16–22 km·h^−1^)	↓ HI in match 2 compared to matches 1 and 3 ↑ impacts in match 3 compared to matches 1 and 2	81.3
Soroka and Lago-Penas [44]	301 elite players playing in the 2014 World Cup (FB *n* = 59, CD *n* = 57, CM *n* = 61, WM *n* = 56, FWD *n* = 68)	Semi-automated video system (ProZone)	3 successive matches (4 days between matches 1 and 2 and, 2 and 3)	TD and distance covered in each locomotive activity Sprint (> 23.1 km·h^−1^) HIR (19.1–23.0 km·h^−1^) MIR (14.1–19.0 km·h^−1^) Walking and light-intensity (0.0–14.0 km·h^−1^)	↑ TD in match 3 compared to matches 1 and 2 ↑ walking and light intensity and MIR in 1^st^ half of match 3 compared to matches 1 and 2 ↑ TD and HIR in match 1 compared to match 3 for CM ↑ TD in match 2 compared to match 1, ↑ MIR in match 3 compared to match 2, ↑ HIR in match 3 compared to matches 1 and 2 for WM ↑TD in match 3 compared to match 2 for FWD	85.7
Penedo-Jamardo et al. [15]	4491 player observations across 18 German clubs (FB *n* = 1079, CD *n* = 1141, CM *n* = 1118, WM *n* = 593, FWD *n* = 560)	Semi-automatic optical tracking system (VISTRACK)	306 matches with comparisons between recovery cycles < 4, 4–5 and > 5 days between matches during early, mid and late season macrocycles. Plus, microcycles with 3- and 4-days recovery	TD, frequency of fast runs and sprints Sprint (> 4.0 m^.^s^−1^ for ≥ 2 s and > 6.3 m^.^s^−1^ for ≥ 1 s) Fast runs (> 5.0 m^.^s^−1^ for ≥ 1 s)	↓ TD with recovery cycle < 4 days Main effects for positional role and period of the season ↓ TD with recovery cycle < 4 days compared to 4–5 and > 5 days recovery for CD, during the mid and late season, respectively ↓ TD with recovery cycle < 4 days compared to > 5 days regardless of macrocycle and ↓ fast runs during the late season for FB. FB also covered less distance 3 days compared to 4 days in mid-and late-season ↓ TD, HIR and sprints when < 4 days during mid-season for WM	85.7
Palucci Vieira et al. [26]	40 professional players from the same Brazilian club	GPS devices (QSTARZ 1 Hz)	1 match vs. 2 successive matches	TD, frequency of HI, maximal sprinting speed, average speed HI (≥ 15 km·h^−1^)	↓ HI for forwards during 2 successive matches All other parameters no effect	92.9
Morgans et al. [37]	21 professional players from the same English club	GPS devices (STATSports)	5 successive matches in 15 days (7 matches in 32 days total)	TD, HIR, sprinting distance Sprint (< 25.0 km·h^−1^) HIR (> 19.8 km·h^−1^)	No effect	73.3
Jones et al. [25]	37 professional outfield players from the same English club	GPS devices (Catapult 10 Hz)	79 matches with comparisons between three congestion scenarios: 1 match vs. 2 matches (< 4 days recovery) vs. 3 matches (< 4 days recovery) per week	TD, distance covered in each locomotive activity, 3D PlayerLoad™ per distance covered (au·m^−1^), PlayerLoad™ anterior–posterior per distance covered (au·m^−1^), PlayerLoad™ medio-lateral per distance covered (au·m^−1^), PlayerLoad™ vertical per distance covered (au·m^−1^) Further measured in 15-min epochs LIR (< 4.0 m·s^−1^) MIR (4.0–5.5 m·s^−1^) HIR (5.5–7.0 m·s^−1^) Sprint (> 7.0 m·s^−1^)	↑ TD in minutes 0–15 and 15–30 during 2 matches vs. 3 matches per week ↑ TD in the 15–30-min period in 1 match vs. 3 matches per week ↑ TD during the 30–45-min period in 2 matches vs. 1 match per week ↓ TD in the 75–90-min period in 3 matches vs. both 1 and 2 matches per week ↑ LIR in the 40–45-min period of 2 matches vs. 1 match per week ↓ LIR in the 75- to 90-min period in 3 matches vs. both 1 match and 2 matches per week ↑MIR during the 0- to 15-min period of 2 matches vs. 3 matches per week ↑ Sprint distance in the 30- to 45-min epoch in 3 matches vs. 1 and 3 matches per week	93.3

*ACC* accelerations, *CAM* center attacking midfielder, *CB* center back, *CDM* central defensive midfielder, *CM* center midfielder, *DEC* decelerations, *FB* full back, *FWD* forward, *HI* high intensity, *HIR* high intensity/speed running, *LIR* low intensity/speed running, *MIR* moderate intensity/speed running, *TD* total distance, *TD/min* total distance per minute, *VHIR* very high intensity running, *WM* wide midfielder

### Quality of Studies

There was good agreement between raters for the quality of studies (*ĸ* = 0.718; 95% CI 0.487–0.949, *p* = 0.0005). The mean methodological quality score for the 17 articles was 74.9 ± 15.7%, with no articles achieving a score of 100% (Table 2). One article scored below 50%, with seven achieving a score between 50 and 75% (good methodological quality) and nine achieving a score over 75% (excellent methodological quality). The criteria that were not met consistently were: criterion 16, related to detailing the limitations of the study; criterion 5, related to justification of sample size; and criterion 7, description of the reliability of the outcome measures.

### Pooled Effect Estimate

**Fig. 2:**
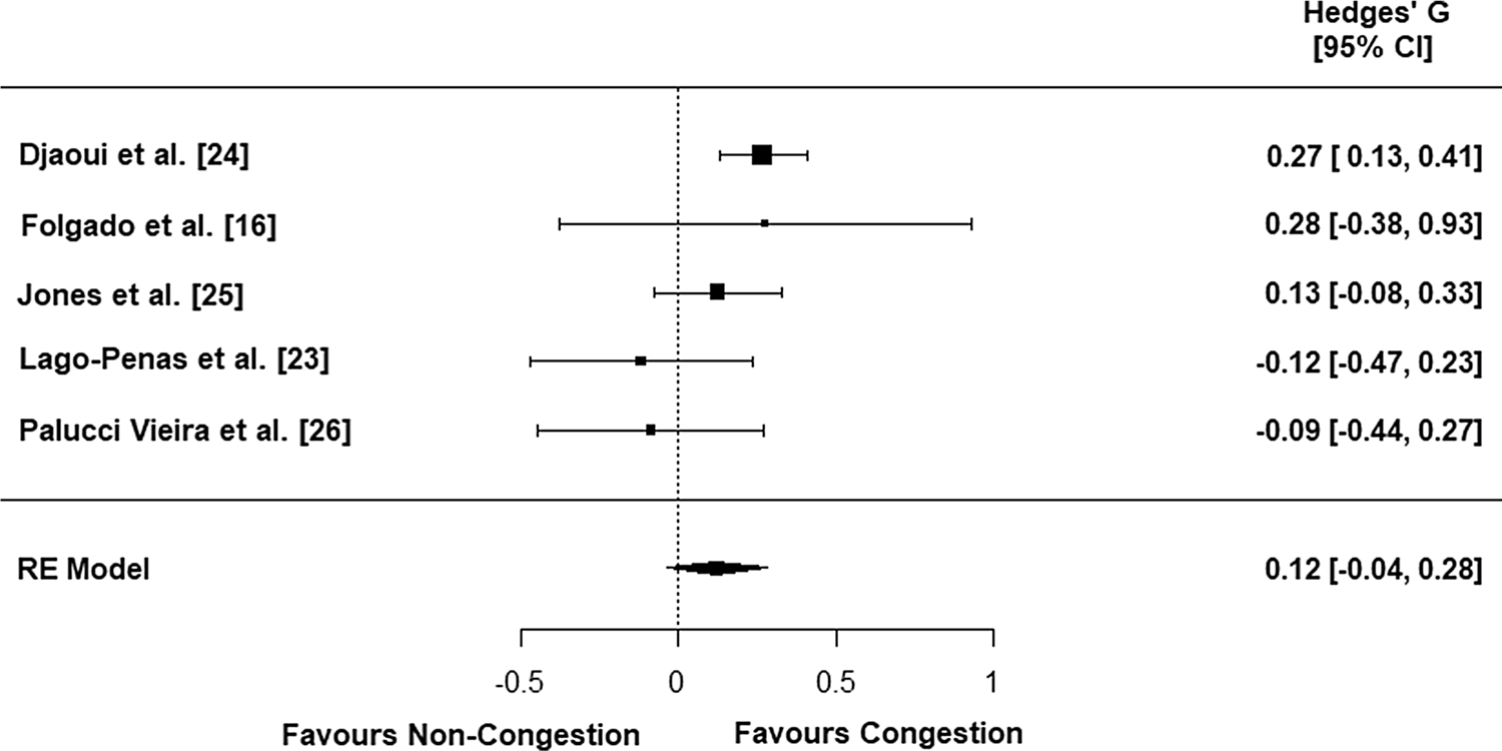
Forest plot of studies meeting inclusion criteria. *CI* confidence interval, *RE model* random effects model

**Fig. 3:**
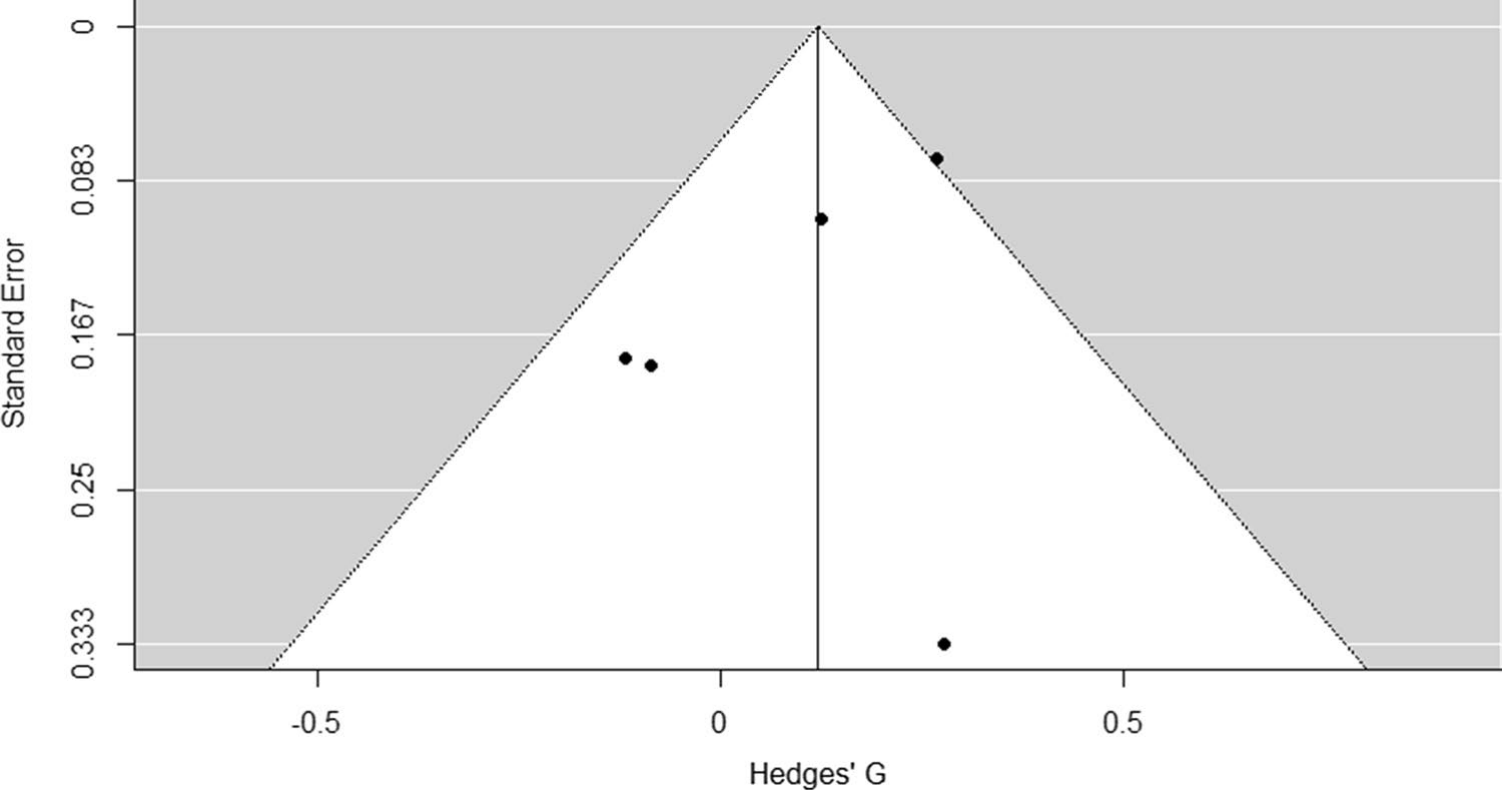
Funnel plot (standard error vs. Hedges’ *G*) for studies meeting inclusion criteria

Results of the meta-analysis revealed no significant effect of fixture congestion on total distance covered (*p* = 0.134), with a trivial effect size [pooled SMD = 0.12 (− 0.04, 0.28); Fig. 2]. Between-study variance, heterogeneity, and inconsistency across studies were moderate [Cochrane’s *Q* = 6.7, *p* = 0.150, *I*^2^ = 40.7% (CI 0.00, 93.34)]. Visual inspection of the funnel plot (Fig. 3) revealed some asymmetry and Duval and Tweedie’s Trim and Fill method identified one missing article from the right side of the plot. When accounting for this missing article, there was a significant effect of fixture congestion on total distance covered (*p* = 0.045) but still with a trivial effect size [pooled SMD = 0.16 (0.00, 0.32)].

## Discussion

### Interpretation of Meta-analysis Findings

We identified no effect of fixture congestion on total distance covered during soccer match-play [*p* = 0.134, pooled SMD = 0.12 (− 0.04, 0.28); Fig. 2]. When all studies were grouped together, distance covered during a congested period was 10,565 ± 991 m and 10,475 ± 880 m during a non-congested period. There were differences between the five studies with regard to the method used to measure distance covered. Three of the studies used semi-automated tracking systems (Amisco: [23, 24] and ProZone: [16]) and two used Micromechanical systems (MEMS) devices (Catapult Sports Optimeye X4: [25] and Qstarz-1 Hz: [26]). Furthermore, in this meta-analysis, the number of player observations was used as the method of sampling. The number of player observations varied between studies, as did the number of observations within studies between congested and non-congested periods (although the sum of player observations between congested and non-congested periods when all five studies were combined was 836 and 820, respectively). Therefore, the differences in equipment used and observation frequency may explain the moderate heterogeneity observed (*I*^2^ = 40.7%). Indeed, researchers have demonstrated that there is small-to-moderate differences in total distance covered when simultaneously measured by both automated tracking systems and MEMS devices during soccer match-play [27, 28]. Therefore, although the present meta-analysis suggests no differences in total distance covered between congested and non-congested periods, further studies should look to use similar methods to measure physical performance, and use consistent movement velocity thresholds when measuring distances covered at different movement intensities. Metrics such as high-intensity distance covered, sprints, accelerations and decelerations, are likely to be of greater interest to practitioners and coaches and, therefore, these measures should be homogeneous between studies where possible.

The low number of articles eligible for the meta-analysis is reflective of an inconsistent methodological approach between studies in this area. We were unable to analyse any other variables, including arguably more relevant outcome measures, such as high-intensity running, sprinting, etc., as studies employed different thresholds when categorising different movements. Furthermore, some studies did not directly compare a congested period to a non-congested period in the same group of players and instead compared the first match in a congested schedule to subsequent matches. This exposed the analysis to the inherent variability evident in professional soccer match-play, due to the stochastic, dynamic nature of the sport [29, 30]. However, that is not to say this same variability may not influence the comparison between a congested and non-congested period, which is dependent on the sample size of the individual study. We identified using Duval and Tweedie’s Trim and Fill method that there was one missing article on the right side of the plot. Thus, when accounting for this missing article, there was a significant effect of fixture congestion on total distance covered (*p* = 0.045) but still with a trivial effect size [pooled SMD = 0.16 (0.00, 0.32)]. This may be due to authors not publishing data that suggest players cover greater distance in a congested fixture period. Nonetheless, we stress that this finding should be interpreted with caution as tests for funnel plot asymmetry tend to only have power to detect true effects when there are ≥ 10 or more articles included in a meta-analysis [22].

### Physical Performance

As highlighted in Sect. 5.1, there appears to have no negative effect of fixture congestion on the total running distance covered by male professional players. However, total distance covered is but one measure of physical performance, and whilst arguably a less relevant one than other measures, is commonly used by practitioners [31]. Notably, the majority of studies included in this systematic review also measured a number of other physical performance metrics in conjunction with total distance covered. However, not only were there methodological inconsistencies between studies for the movement velocity thresholds employed, but there were also differences in how authors compared a congested period to a non-congested period.

Some studies have attempted to assess the physical response to three successive elite soccer matches performed over a 6- to 7-day period [1, 16, 32]. These studies all reported no differences in the total distance covered and distances covered at high intensities (HID) across the successive matches. Folgado et al. [16] also identified no differences in the distances covered in all locomotion categories across the successive matches. However, Odetoyinbo et al. [32] did identify that distance covered and duration of walking, HID whilst in possession of the ball, and HID when the ball was out of play were all significantly lower in the third match compared to the first. These data suggest total distance covered and overall HID are not significantly impaired when three matches are played over 7 days; however, when three matches are performed over 6 days, players may potentially alter their activity profiles in an attempt to reduce the volume of activity performed [32]. However, and critically, it is not known if these observed differences are a result of contextual factors or reduced physical capacity. In contrast to these investigations, Andrzejewski et al. [14] observed significantly higher total distance covered and distances covered in different speed threshold categories up to 21 km·h^−1^ in the third match of three matches in 7 days’ microcycle, with no changes in the number of sprints or distance covered ≥ 21 km·h^−1^. However, the data were from 11 players playing for the same club, with no indication from the authors on the quality of the opposition faced in each match, or the score line. It is possible that the third match was against superior opposition and/or a closer match score-wise compared to the other two matches, which may have influenced the physical response [33, 34].

A strength of Odetoyinbo et al. [32] is that the data collected were from 16 players playing for four different teams, whereas the players from Folgado et al. [16] and Carling and Dupont [1] were from the same team (in the English Premier League and French Ligue 1, respectively). The first two matches in the study conducted by Odetoyinbo et al. [32] were interspersed with 48 h recovery, whereas each of the matches in Carling et al. and Folgado et al. [1, 16] was interspersed by 72 h of recovery. Therefore, it seems feasible that the reduced recovery time associated with the first two matches in Odetoyinbo et al. [32] may have elicited the observed fatigue response identified in the third match. Other authors have compared the physical outputs of players when two matches were played with 3 days’ rest in between [35]. There was no difference between matches played in close proximity by elite Spanish players [35]. However, this study scored 40% on the quality assessment tool (low quality; Table 2) and did not report how many matches were included in the study, or any contextual factors, such as match location, quality of opposition, and tactical approach. Furthermore, Dupont et al. [36] observed no differences in physical performance when elite French players played two matches in a week. However, these authors did not report any data within their manuscript, making comparisons to other studies difficult.

Studies conducted by Carling et al. [2] and Dellal et al. [3] assessed the physical response to a period of prolonged fixture congestion (six–eight matches performed over 18–26 days) in elite French soccer. Dellal et al. [3] identified no significant differences in any of the physical performance measures recorded across the six congested matches; however, any statistically significant differences between individual matches may have been missed by a lack of an overall main effect. Although the authors compared the data collected during the periods of fixture congestion to that identified during a non-congested schedule, this was only for injuries not physical performance. Therefore, it would have been pertinent for the authors not only to compare physical performance within a congested period (e.g., match 1 compared to match 6), but also compare to a non-congested period in the same group of players. In contrast, Carling et al. [2] identified that distances covered at low intensities and total distance covered differed between some matches in an eight-match congested schedule. However, this was not systematic, with one match in particular (match 4) being significantly different to five other matches, and matches 7 and 8 being different to two matches and one match, respectively. However, when compared with periods of no congestion (although the authors did not define what this was), there was no difference in any of the physical performance metrics measured, indicating that this group of elite French players was able to maintain physical output during a congested schedule. However, it should be noted that the authors did not report how many of the players who were included in the congested analysis played in the non-congested matches, including the number of minutes played. Therefore, caution should be taken when interpreting the findings of this study.

Morgans et al. [37] followed a similar methodology, assessing physical performance changes during seven matches in 29-day microcycle in a group of English Premier League players. Whilst the authors reported the overall sample size (*n* = 21), they did not report how many players played in all seven matches, or the percentage that played > 75 min. Therefore, the findings may have been affected by substitutions and players not starting or playing in all of the matches.

Mohr et al. [38] took a different approach to most of the other studies reviewed, as instead of using data from professional soccer match-play, they created three matches in one-week scenario in a group of competitive male players (*n* = 40; had to have played in the top three divisions of their country’s league system in the past 5 years; the country is not specified). The authors observed a 7–14% decrement in high-intensity distance covered in the second match compared to the first (played 3 days prior) and third (played 4 days after) matches. No other differences were observed between matches, and this difference in high-intensity distance is lower than the coefficient of variation previously reported for this measure [29, 30] and, therefore, may be reflective of match-to-match variability as opposed to residual fatigue from the first match. Although beyond the scope of this systematic review, these authors showed that players were unable to fully recover physical function between the three matches, and that there was an increase in muscle soreness and muscular inflammation, particularly following the second match. This was less pronounced following match three, which may demonstrate that there is a significant effect on performance between 3 and 4 days of recovery.

All studies included in the meta-analysis also reported data from other measures of physical performance, not just total distance covered. Both Folgado et al. [16] and Lago-Peñas et al. [23] observed no changes in distance covered at various velocity ranges between a congested and non-congested period. It should be noted that the six matches in Folgado et al. [16] were all played (and won) at home against lower level opposition, which may have influenced the observed response [33]. Similarly, Djaoui et al. [24] observed no differences in distance covered at speeds ≥ 18 km·h^−1^ between congested and non-congested periods, although they showed central defenders cover more low-intensity (< 12 km·h^−1^) distance during congested periods. It is well established that position-specific differences in physical performance exist during soccer match-play [34, 39–41] and, as such, match-play analyses should be considered in relation to player positions. These positional differences also exist during periods of fixture congestion [15, 24, 42]. In support of this, Carling et al. [43] identified that defensive players were more likely to complete > 75 min of match-play compared to other positions, thus exposing defensive players to congested schedules. Whilst low-intensity distance covered was significantly increased in central defenders in Djaoui et al. [24], the distance covered by central defenders at higher velocities, whilst not statistically different, was lower in the congested periods. Furthermore, Penedo-Jamardo et al. [15] reported significantly lower distances covered and number of fast runs (speed of ≥ 5.0 m‧s^−2^ for ≥ 1 s) performed by central defenders during matches preceded by < 4 days recovery from a previous match, compared to > 5 days.

Therefore, this may indicate a change in movement intensity by central defenders during fixture congestion, either by a conscious pacing strategy, or due to match-related fatigue. However, Jones et al., Palucci Vieira et al. and Soroka and Lago-Penas [25, 26, 44] did not observe any changes in central defensive players’ physical performance in congested periods. In professional Brazilian football players, fixture congestion has differential effects on physical performance [26]. Palucci Vieira et al. [26] observed position, formation, match location and match outcome-specific effects during congested periods (defined as two matches a week vs. one match a week) on some physical performance parameters. In particular, they showed that forwards perform less high-intensity activity in congested periods and there is less high-intensity activity in drawn matches and when using a 4-3-3 formation as opposed to 4-4-2. Furthermore, total distance and average velocities were reduced during congested fixtures played away compared to at home. However, it must be noted that all effect sizes for these reported differences were trivial or small [26].

Soroka and Lago-Penas [44] analysed players who completed 90 min of three matches each separated by 4 days of rest in the group stage of the 2014 FIFA World Cup. They found that players covered more distance in the third match than the second match (and the first match compared to the second match), with concomitant increases in the amount of light-intensity and moderate-intensity running in the first half of the third match compared to both the first and second matches. This may be reflective of the importance of the final group stage match, although no differences were observed for high-intensity distance or number of sprints. These authors also observed position-specific changes in physical performance during the three group stage matches, with central midfielders covering less total distance and high-intensity running distance during the third match compared to first match, whereas wide midfielders and forwards covered more total distance and wide midfielders also covered more distance at moderate and high intensities. Without contextual data, such as the formations employed by teams in the final group stage matches, or the permutations regarding qualification to the knockout stage, it is difficult to fully interpret these findings.

Penedo-Jamardo et al. [15] observed significantly lower distance covered by full-backs and wide midfielders (dependent on season phase) when matches were separated by < 4 days compared to ≥ 5 days. Furthermore, these authors observed reduced total distance covered in the early- and mid-season phase of the 2011/12 German Bundesliga season when there were < 4 days of recovery between matches compared to > 5 days recovery, irrespective of playing position. With the high number of matches (*n* = 306) and player observations (*n* = 4491) in this study, the findings may indicate that less than 4 days of recovery between matches are insufficient for players to be able to maintain some aspects of physical performance (see Table 2). However, the number of fast runs and sprints was not affected by fixture congestion. The findings of this study are in contrast to the findings of the meta-analysis (Sect. 4.2), and indicate fixture congestion does indeed have a negative impact on performance.

Whilst Jones et al. [25] did not observe any differences in physical performance during fixture congestion when players were separated by position, they did observe reductions in total, low-intensity, and moderate-intensity distance covered in specific 15-min epochs in the final match of three matches in a week’s microcycle compared to when matches were played in one match per week or two matches per week microcycle. This is particularly relevant as when they compared whole match averages, there were no differences between matches in a congested vs. non-congested period. The findings from Penedo-Jamardo et al. and Jones et al. [15, 25] seem to suggest that reductions in low-intensity distance covered when there is limited recovery time between matches may be due to conscious or unconscious pacing strategies employed by the players to preserve their ability to perform high-intensity movements [25, 45].

### Technical and Tactical Performance

In comparison to the larger body of literature that has investigated changes in physical performance during periods of fixture congestion, there is a paucity of research that has examined changes in technical (i.e., skill) and tactical performance. Within our searches, we identified five published journal articles that have analysed the impact of fixture congestion on technical (four) or tactical (one) performance (Table 3). Technical performance is well maintained during periods of fixture congestion, with no changes in performance during a microcycle when players are exposed to three matches in 7 days or less [14], or when six consecutive matches are played with 3 days’ rest in between [1]. The findings of these two studies should be interpreted with caution, as the matches may have been influenced by contextual factors (e.g., match location, quality of opposition, and score line) and the small, homogenous sample sizes. Indeed, Andrzejewski et al. [14] investigated 11 players from the same Polish Ekstralasa (highest professional division in Poland) club, and Carling & Dupont [1] assessed seven midfield players who either played in one (four players) or two (three players) sequences of three matches in 7 days during 1 month of the French Ligue 1 season. However, two studies with larger sample sizes and a higher number of instances of fixture congestion have also identified no effect of fixture congestion on technical performance [3, 15]. Nevertheless, Penedo-Jamardo [15] only investigated the effect of time between matches on one variable (pass accuracy), with no indication of how this was measured, including the validity and reliability of the measure. Furthermore, in the three instances of fixture congestion analysed in Dellal et al. [3], only five or six players’ technical performance was assessed in each instance, with all players representing the same French Ligue 1 club. Again, performances may have been influenced by contextual variables and be reflective of this club only (as acknowledged by the authors). As such, whilst the current evidence suggests that fixture congestion has no effect on technical performance, further investigations utilising data from multiple clubs with an analysis of position-specific differences and a broader range of more meaningful measures (e.g., expected goals for and against, expected assists, pass/cross accuracy in the final third of the pitch, and loss or gain of possession due to interceptions). As technical performance between matches has been shown to be more variable than physical performance [46], large datasets are required to ensure any differences during congested schedules are meaningful and reflective of actual changes.

**Table 3:** Summary of studies investigating technical and/or tactical performance during periods of fixture congestion

References	Participants	Match data collection methods	Fixture congestion scenario	Outcome measures	Main findings	Quality score (%)
Carling and Dupont [1]	7 professional midfield (central and wide) players playing for the same French Ligue 1 club	Semi-automated video system (Amisco)	3 successive matches in 7 days or less	Total number of passes, percentage of completed or uncompleted passes, number of ball possessions and possessions gained or lost, number of touches per possession, number of duels and percentage of duels won or lost	No effect	71.4
Andrzejewski et al. [10]	11 professional players playing for the same Polish Ekstralasa (highest tier) club	Semi-automated video system (Amisco)	1 vs 2 match weekly microcycles	Total individual ball possession, contacts with the ball, passes, ground challenges and aerial challenges	No effect	60.0
Dellal et al. [3]	16 professional outfield players from the same French Ligue 1 club	Semi-automated video system (Amisco)	3 instances of six consecutive matches separated by 3 days. Five players in the first instance, six in the second instance and five in the third instance	Percentage of successful passes, number of balls lost, total number of touches per possession and percentage of duels won	No effect	93.3
Folgado et al. [12]	23 professional outfield players from the same English Premier League club	Semi-automated video system (ProZone) and Hilbert Transform	3 successive matches with 3 days between matches	Space–time synchronisation between pairs of players and player displacement on horizontal and vertical axes	↓ synchronisation during periods of fixture congestion at low and moderate movement intensities (0–3.5 km·h^−1^ and 3.6–14.3 km·h^−1^). No differences at high movement intensities (> 14.4 km·h^−1^)	60.0
Penedo-Jamardo et al. [11]	4491 player observations across 18 German clubs (Bundesliga) (fullbacks *n* = 1079, central defenders *n* = 1141, central midfielders *n* = 1118, wide midfielders *n* = 593, attackers *n* = 560)	Semi-automatic optical tracking system (VISTRACK)	306 matches with comparisons between recovery cycles < 4, 4–5 and > 5 days between matches during early, mid and late season macrocycles. Plus microcycles with 3 and 4 days recovery	Percentage of successful passes	No effect	85.7

↓ and ↑ denote significant reductions and significant increases in measures respectively

Only one published research investigation has assessed changes in tactical performance during a period of fixture congestion. Folgado et al. [16] assessed dyadic synchronisation of pairs of players in an English Premier League team during a period of congested (three matches with 3 days recovery between each match) vs. non-congested fixtures (three matches with 6 or more days recovery between each match). The authors observed reduced synchronisation between dyads [in particular between wide players (i.e., full-backs and wingers) and other positions] during the congested period vs. the non-congested period at low/moderate movement intensities (0.0–14.3 km·h^−1^), but not at high/very high movement intensities (> 14.4 km·h^−1^). They postulated that the reduced synchronisation at low/moderate intensities may have been due to mental fatigue, and players deliberately adopting pacing strategies to preserve energy [17, 45]. Nevertheless, these changes in synchronisation during a congested period may also be due to the lower amount of available time to train between matches, with likely greater emphasis placed on rest and regeneration protocols. With less time to train, there is less opportunity for teams to train together and improve tactical behaviours. It should be noted that all matches were played (and won) against lower level opposition, which may have influenced the observed response (e.g., players ‘switching off’ when leading or playing against perceived lower level opposition). Nonetheless, the de-synchronisation between specific dyads may expose teams to counterattacks, where the suboptimal spatial and temporal relationship between players allows opponents opportunities to attack, particularly through wide areas. However, further research on tactical performance changes during fixture congestion is required, with larger sample sizes (e.g., multiple teams) and a greater number of instances of congestion.

### Future Research Directions and Recommendations

Whilst the journal articles discussed provide somewhat of an overview of the effect of fixture congestion on performance, there is scope for future research to improve methods employed and expand the currently available data. Studies that do not compare a congested period to a non-congested period in the same group of players should be avoided, as comparing within a single congested microcycle only elicits a high risk of bias due to match-to-match variability, and leaves the measured variables open to contextual factors. Furthermore, to allow for future meta-analyses on other performance variables, such as high-intensity running, sprint speed, and the number of accelerations and decelerations, studies should aim to employ the same threshold definitions to allow for data to be accurately analysed and compared across studies, as well as report temporal changes across matches (e.g., across 15-min periods; [25]). Additionally, and in line with a call for more transparent research practices [47], we encourage authors to make their data available for analysis (whilst accounting for participant anonymity) on platforms such as the Open Science Framework (osf.io), as we have done in this article.

Assessing the types of movement performed would also provide a clearer picture of the effect of fixture congestion. For example, are players changing how much they press the opposition during congested periods, and how much of their movement contributes to overall attacking sequences? A recent mixed-method study [48] used a combination of network analysis and qualitative content analysis to assess the attacking behaviour of AS Monaco players during the 2016/17 French Ligue 1 season. Through interviews with the head coach and performance analyst, the authors were able to identify *why* certain players performed the way they did during the season. This type of collaboration within the context of fixture congestion would provide a robust overview of how performance changes during congestion, and how coaches potentially manipulate their tactics in the face of a high number of matches in a short duration [49].

The most recent paper assessing the frequency of exposure to fixture congestion was published in 2015 and only analysed players from one club [13]. In the context of contemporary fixture scheduling and statistical power, this article requires an update, with more than one club’s exposure to fixture congestion assessed. Furthermore, no studies have investigated the impact of fixture congestion in female soccer players; whilst this may not be a particularly prevalent issue during domestic competition schedules, the FIFA Women’s World Cup and the UEFA Women’s Championship may expose female players to congested periods that they are not accustomed to. Therefore, assessing the impact of fixture congestion on female players is required, especially as physical performance and markers of inflammation have been shown to change negatively following match-play in elite female soccer players [50, 51].

As players cover more high-intensity distance when playing superior opposition [52], if a team is to play three matches in 6–7 days all against better-ranked teams, there may be an exacerbated fatigue response in the recovery phase as players will have a higher physical output. This may then influence potential injury risk. Therefore, practitioners should aim to assess recovery daily during periods of fixture congestion to assess which players may be at highest risk of reduced performance and injury. Additionally, matches that require extra-time are typically played during congested periods (e.g., on a midweek evening between two weekend league matches, or during the knockout phase of international tournaments). Case studies have shown that ET may have an additional negative impact on recovery [53, 54]; however, studies in controlled environments (i.e., using laboratory-based simulations) are required.

In support of Page et al. [55], laboratory-based soccer simulations may also help identify the mechanisms that potentially explain reductions in performance during fixture congestion. Likewise, the use of protocols, such as the Intermittent Soccer Performance Test [56], that are performed on non-motorised treadmills and, therefore, can identify changes in running distance/speed could further enhance our understanding of congested match schedules. Mohr et al. [38] assessed the impact of three matches in 1 week and were able to measure recovery every day during that period. However, the design was susceptible to inherent match-to-match variation and, therefore, the use of validated and reliable simulations can increase the robustness of the data [55, 57]. Moreover, studies can then also use such designs to investigate the effectiveness of interventions that accelerate recovery and improve performance during congested periods [18].

### Practical Applications

Coaches and practitioners should be aware that congested fixture periods may have an impact on the physical, technical and tactical performance of players. Whilst tactical performance has only been assessed in one study, there was reduced synchronisation between players, which could negatively impact the tactical strategy implemented. Furthermore, during fixture congestion, there is less high-intensity activity when employing a 4-3-3 formation compared to a 4-4-2 formation [26]. Therefore, coaches may want to identify systems and players that are particularly susceptible to fixture congestion, and adapt their strategies accordingly. For example, as Folgado et al. [16] identified increased susceptibly to counterattacks in wide areas, coaches may want to ensure that defensive midfield players are able to cover and prevent counterattacks in these areas when their team is attacking. However, it should be noted that time to work on tactical behaviours is limited during congested periods, and players may not be able to process complex information in close proximity to matches due to match-induced mental fatigue [58]. The data reported in this review suggest that central defenders in particular are the positional group most exposed to periods of fixture congestion, with attacking players the least exposed due to substitutions and rotation. Whilst central defenders typically have the lowest external workload during matches [34, 40, 59], practitioners should ensure that recovery protocols for these players are optimised and adapted to reflect their greater exposure to match-play compared to some of their teammates. Nevertheless, regardless of playing position, if a player is exposed to repeated match-play during congestion, then it is likely that they will have an increased risk of injury [60] and modulate the intensity of their movements, potentially impacting overall performance.

## Conclusion

The results of the meta-analysis suggest that total distance covered is not impacted by fixture congestion. However, no other variables were assessed quantitatively due to methodological differences between studies, and there was a moderate level of heterogeneity between the five included studies. Nevertheless, some studies have identified a negative effect of fixture congestion on variables, such as low- and moderate-intensity distance covered; this may suggest that players consciously employ pacing strategies to maintain high-intensity actions. Furthermore, this may be position-specific and related to the time in a match. Whilst physical performance is crucial to overall success in soccer, technical and tactical performance are perhaps even more important, and there is a lack of data on these two elements of performance during fixture congestion. In conclusion, fixture congestion is a very contemporary issue, one that players are particularly conscious of [4]. With ever increasing numbers of competitive matches scheduled, more research needs to be conducted using consistent, sensitive measures of performance, including physical, technical and tactical aspects.

## Declarations

### Availability of data and material

Data used in the meta-analysis can be found publicly at the following link: https://osf.io/2q6aj/
